# Cross-Cultural Comparison of Perceived Stigma in Major Depressive Disorder: The Role of Spiritual Well-Being, Resilience, and Quality of Life in Austria and Japan

**DOI:** 10.1177/00207640261473300

**Published:** 2026-08-06

**Authors:** Nataliia Maronchuk, Kie Nomoto, Maya Shirakura, Sota Tomiyama, Jean Lillian Paul, Fabienne Post, Ellen B. Rubinstein, Timo Schurr, Yuya Mizuno, Franziska Tutzer, Hiroyuki Uchida, Alex Hofer

**Affiliations:** 1 Department of Psychiatry, Psychotherapy, Psychosomatics and Medical Psychology, Division of Psychiatry I, Medical University Innsbruck, Austria; 2 Department of Neuropsychiatry, Keio University School of Medicine, Tokyo, Japan; 3 Department of Psychiatry, Tokyo Musashino Hospital, Japan; 4 College of Science, Virginia Polytechnic Institute and State University, Blacksburg, USA; 5 Department of Sociology and Anthropology, North Dakota State University, Fargo, USA; 6 Department of Psychosis Studies, Institute of Psychiatry, Psychology & Neuroscience, King’s College London, UK; 7 East London NHS Foundation Trust, UK

**Keywords:** stigma, major depressive disorder, spiritual well-being, resilience, quality of life, personal and social performance

## Abstract

**Background:**

Perceived stigma in Major Depressive Disorder (MDD) is a well-established barrier to help-seeking, social functioning, and quality of life. However, little is known about how stigma is shaped by cultural context and which psychosocial resources may buffer its effects.

**Aims:**

This study aimed to examine the relationship between perceived stigma and key protective factors – spirituality, resilience, quality of life, and social functioning – in patients with MDD from Austria and Japan.

**Method:**

A total of 101 patients diagnosed with MDD (51 from Austria, 50 from Japan) completed validated measures of perceived stigma, spirituality, resilience, quality of life, and social functioning. Correlational analyses, multiple regression, and interaction term models were used to assess the predictive value of psychosocial variables and country moderation effects.

**Results:**

Spirituality and resilience were negatively associated with perceived stigma across both samples, though only spirituality significantly predicted stigma in regression analyses, increasing to 48% after controlling for age and depression severity. Country-specific models revealed a stronger predictive power in the Japanese sample. While stigma correlated strongly with reduced quality of life in both groups, its association with social functioning varied by stigma dimension and country. The relationship between resilience and financial insecurity was stronger among Japanese study participants than among Austrian participants.

**Conclusions:**

In summary, spirituality emerged as a central protective factor against stigma in MDD patients, particularly in Japan, underscoring its cultural significance. Additionally, our findings reveal a context-specific relationship between economic stress and psychosocial coping strategies. Despite differences in how stigma is expressed across countries, its adverse impact on quality of life was universal. These results suggest that culturally informed approaches can effectively reduce stigma associated with depression.

## Background

Major Depressive Disorder (MDD) affects over 280 million people worldwide, accounts for 4.3% of the global disease burden and is one of the leading causes of disability worldwide (World Health Organization, 2021). Perceived social support and social stigma are thought to play a particularly important role for those affected (Griffiths et al., 2011; Livingston & Boyd, 2010; Wang et al., 2018). Understanding the key factors associated with perceived stigma in MDD across different cultures is essential for the development of targeted mental health interventions.

Growing evidence indicates that spiritual well-being and resilience serve as important protective factors in MDD, being associated with reduced symptom severity, improved coping, and lower perceived stigma (Hofer et al., 2022; Koenig, 2015; Southwick et al., 2014; Tutzer et al., 2024). While spirituality broadly refers to an individual’s search for meaning, connectedness, and transcendence – often encompassing religious and existential dimensions (Koenig, 2015; Pargament, 2011) – spiritual well-being reflects the subjective experience of peace, purpose, and harmony derived from these beliefs (Peterman et al., 2002). A study by Dewi and Hamzah (2019), for example, demonstrated a positive correlation between spirituality and psychological resilience and, as a result, an improved ability of individuals to cope with depressive symptoms. In addition, higher levels of spirituality have been associated with better quality of life (QoL; Borges et al., 2021; Sawab et al., 2024) and lower levels of stress and anxiety (Kioulos et al., 2015; Koenig, 2015; Mizuno et al., 2018). Similarly, resilience, that is, the capacity to recover from adversity, has been linked to an improved QoL (Hofer et al., 2017; Pardeller et al., 2020; Post et al., 2018), with social support and spirituality potentially acting as mediating factors (Chen et al., 2023). In addition, resilience plays a crucial role in mitigating the effects of stigma. Research involving individuals with serious mental illnesses demonstrates that higher resilience correlates with lower self-stigma and greater resistance to stigma (Hofer et al., 2019; Post et al., 2021), indicating that resilient individuals are less likely to internalize negative societal views.

Perceived stigma, understood here as the experience of social exclusion, financial insecurity, internalized shame, and social isolation, is known to negatively affect QoL and functioning in individuals with serious mental illnesses (Alqahtani & Pringle, 2024; Cerit et al., 2012; Hawke et al., 2013; Hawke et al., 2014; Ociskova et al., 2023; Post et al., 2018). Importantly, stigma is not experienced uniformly across cultures. Cross-cultural research has shown marked variation in public attitudes and internalized stigma, with studies from Japan reporting particularly strong associations between mental illness and shame, leading to social withdrawal and delayed help-seeking (Ando et al., 2013). Clearly, Western countries like Austria also face challenges related to stigmatization, though the association between mental illness and shame appears to be weaker there than in East Asian societies (Hofer et al., 2016; Krendl & Pescosolido, 2020). Recent studies in Austria have shown that self-stigmatization is associated with lower rates of seeking help (Zeldovich et al., 2025), and international evidence suggests that stigma is consistently linked to poorer QoL (Juergensen et al., 2024). Although public awareness is improving, stigma remains a barrier to accessing mental healthcare (Nowotny, 2020; Nowotny et al., 2025).

Differences in social norms and support systems may influence stigma experiences in Austria that are distinct from those observed in Japan. These cultural contrasts make Austria and Japan suitable case examples for examining how perceived stigma in MDD may be shaped by broader sociocultural orientations, which is the basis for the present study.

According to cross-cultural research, coping resources may function differently depending on the cultural context. In collectivistic societies, such as Japan, coping strategies are often relational and embedded in communal or spiritual frameworks that emphasize harmony and interdependence (Kim et al., 2008; Markus & Kitayama, 2014). Spiritual well-being may therefore serve as a salient protective factor, offering meaning and connectedness that buffer the effects of stigma (Koenig, 2015; Ma, 2020). In individualistic societies like Austria, where self-reliance is emphasized, resilience – the ability to adapt to adversity – may play a stronger role in reducing stigma. This is consistent with studies showing its buffering effect in Western settings (Corrigan et al., 2006; Hofer et al., 2016; Southwick et al., 2014).

## Aims

To our knowledge, the effects of spiritual well-being and resilience on perceived stigma in MDD have not previously been directly tested across different cultural contexts. Therefore, the comparison between Japan and Austria provides a unique opportunity to explore shared and culture-specific mechanisms underlying stigma in depression. We use country as a pragmatic unit of comparison, recognizing that cultural norms and healthcare contexts may shape stigma experiences. To this end, we examined the relationship between perceived stigma and several psychosocial factors – spiritual well-being, resilience, QoL, and personal and social functioning – among individuals with MDD in Austria and Japan.

We formulated three hypotheses: H1: Across both countries, higher levels of spiritual well-being and resilience are associated with lower perceived stigma, and higher stigma is linked to poorer QoL and social functioning. H2: The strength of these associations varies by country, with spiritual well-being playing a stronger protective role in Japan and resilience showing a stronger association with lower stigma in Austria. H3: Spiritual well-being, resilience, QoL, and social functioning are significant predictors of perceived stigma in both countries.

## Method

### Participants and Procedures

The study sample consisted of individuals with MDD according to the Diagnostic and Statistical Manual of Mental Disorders (DSM-5; American Psychiatric Association, 2022) ⩾ 18 years of age and with a maximum psychiatric treatment duration of 10 years. Study participants were recruited from in- and outpatient mental health services in Innsbruck, Austria and Tokyo, Japan. Individuals with any physical or neurological illness or any condition affecting neural or cerebrovascular function were excluded. Sample size calculations were conducted with G*Power (version 3.1.9.2) and based on the assumption of a type-one error probability of *α* = .05 and a power of 1−*β* = .8. Concerning correlation analyses with two-tailed hypothesis testing, a sample size *N* of 100 patients is sufficient for detecting a correlation coefficient *r* of .272. A consecutive sampling strategy was employed, whereby all eligible patients meeting the inclusion criteria during the recruitment period were invited to participate.

The study was approved by the ethics committees of the Medical University Innsbruck (approval number 1268/2023) and the Institutional Review Board of Keio University Hospital (approval number 20211034). Participation was voluntary and did not affect the care patients received. All those who agreed to participate signed informed consent forms.

### Assessments

The questionnaires used at each center were in German or Japanese language. In addition to collecting sociodemographic and clinical data, trained psychiatrists and psychologists assessed psychopathological symptoms using the Montgomery Åsberg Depression Rating Scale (MADRS; Montgomery & Åsberg, 1979; Schmidtke et al., 1988; Takahashi et al., 2004). This scale consists of 10 items (apparent sadness, reported sadness, inner tension, reduced sleep, reduced appetite, concentration difficulties, lassitude, inability to feel, pessimistic thoughts, and suicidal thoughts) with each item yielding a score of 0 to 6. Accordingly, the overall score ranges from 0 to 60, with higher scores reflecting more severe depression.

We assessed perceived stigma using the 24-item Social Impact Scale (SIS, Eichhorn et al., 2015; Fife & Wright, 2000).

Based on modified labeling theory, the SIS was initially developed to assess stigma in people with HIV/AIDS or cancer. It measures four dimensions – social exclusion, financial insecurity, internalized shame, and social isolation – using a 4-point Likert scale (1 = “strongly disagree” to 4 = “strongly agree”). Total scores range from 24 to 96, with higher scores indicating a stronger sense of stigmatization. Due to a procedural error, item 24 of the questionnaire was missing in the Austrian sample. To address this, the mean of the respective subscale (social isolation) was imputed for the missing item, ensuring comparability across samples while minimizing data loss. This is a common approach to handling missing items in psychometric scales when internal consistency is high (Little & Rubin, 2019; Schafer & Graham, 2002). The affected subscale (social isolation) demonstrated good internal consistency (*α* = .84), indicating that the items capture a coherent construct and that the imputed value is unlikely to substantially bias the results. Previous research suggests that replacing a single missing item within a reliable subscale with the mean has a minimal impact on the scale’s properties and is considered acceptable when the proportion of missing data is low (Graham, 2009). Therefore, this procedure is unlikely to compromise the robustness of the social isolation subscale in the present study. As no validated Japanese version of the SIS was available, forward-backward translation procedure was undertaken. The scale was translated into Japanese by the Japanese team members and subsequently back-translated into English by other members. The back-translated version was reviewed by one of the original authors of the SIS (E. Wright, personal communication), who confirmed that it was highly comparable to the original version. Minor wording modifications were suggested for two items in the social exclusion subscale to better reflect the temporal nuance of the original items; these modifications were incorporated into the final Japanese version.

We assessed spiritual well-being using the 12-item Functional Assessment of Chronic Illness Therapy – Spiritual Well-Being Non-Illness Version (FACIT-Sp NI; www.facit.org; Peterman et al., 2002). The three-factor model was used due to its psychometric robustness, better applicability, and consistency across different populations, particularly in relation to mental health (Canada et al., 2008; Murphy et al., 2010). There are four questions related to each of the following factors: sense of meaning in life, peacefulness, and sense of strength and comfort from one’s faith. Each item is scored from 0 (“not at all”) to 4 (“very much”) and higher scores indicate greater spiritual well-being.

Psychological resilience was measured using the 25-item Resilience Scale (RS; Nishi et al., 2010; Schumacher et al., 2005; Wagnild & Young, 1993). This scale conceptualizes resilience as “a positive personality characteristic that enhances individual adaptation.” Each item is scored from 1 (“strongly disagree”) to 7 (“strongly agree”), and total scores range from 25 to 175 with higher scores indicating higher resilience.

Health-related QoL was assessed by means of the short version of the World Health Organization (WHO) QoL questionnaire (WHOQOL-BREF; Whoqol Group WHO, 1998). This instrument consists of 26 questions scored in the domains of overall satisfaction with QoL, physical health, psychological health, social relationships, and environment. Each question is rated on a 5-point scale, domain scores are transformed to lie between 0 and 100.

Social functioning was assessed using the Personal and Social Performance Scale (PSP; Inada et al., 2011; Juckel et al., 2008; Morosini et al., 2000). This 100-point single-item scale measures patients’ functioning in four domains: socially useful activities, personal and social relationships, self-care, and disturbing and aggressive behavior. Higher scores indicate higher functioning.

### Statistical Analyses

All analyses were conducted using *IBM SPSS Statistics for Windows, Version 29.0.0* (IBM Corp., 2022). Prior to analysis, the assumptions underlying parametric tests were examined. The distribution of continuous variables was assessed using visual inspection of histograms and Q-Q plots, as well as skewness and kurtosis statistics. We found that the variables were approximately normally distributed. For the multiple regression analyses, the assumptions of linearity, homoscedasticity, independence of residuals, and the absence of multicollinearity were evaluated and found to be acceptable. Group comparisons were performed using Pearson’s chi-squared test for sociodemographic variables, Student’s *t*-test for clinical scores, and the Mann–Whitney *U* test for duration of illness due to its non-normal distribution. To adjust for multiple testing, Benjamini–Hochberg corrected *p*-values are reported. A two-tailed *p* < .05 was considered statistically significant.

Effect sizes were interpreted according to Cohen’s guidelines: for mean comparisons, *d* ⩾ 0.2 (small), *d* ⩾ 0.5 (medium), and *d* ⩾ 0.8 (large); for correlations, *r* = .10–.29 (small), .30–.49 (medium), and ⩾ .50 (large) (Cohen, 2013). Pearson’s product-moment correlation coefficients (*r*) were calculated to examine the associations between the variables, and Fisher’s *r*-to-*z* transformation was applied to test the differences between the correlation coefficients across the countries. Variance explanation was classified as weak/low (*R2* = .02), moderate (*R*^2^ = .13), or strong/high (*R2* = .26) (Cohen, 2013).

Multiple regression analyses were conducted to examine the predictive role of spiritual well-being, resilience, QoL, personal and social performance, and country on perceived stigma. Standardized coefficients were reported for interpretation. A hierarchical multiple regression was performed to account for significant sample differences by adding age and MADRS depression severity in a second block. The overall sample size (*N* = 101) was considered adequate for the planned regression analyses, providing approximately 14 to 17 observations per predictor in the main models, which exceeds commonly recommended minimum ratios for multiple regression (Babyak, 2004). Nevertheless, the study was primarily exploratory and may have been underpowered to detect small effects or subtle interaction terms.

For reliability analyses, Cronbach’s alpha was used to evaluate the internal consistency of the full 24-item Social Impact Scale (SIS) and the 7-item social isolation subscale, which included mean imputation for one missing item. Internal consistency was excellent for the full SIS (*α* = .91) and high for the social isolation subscale (*α* = .84) (Blanz, 2021).

## Results

### Descriptive Statistics

A total of 101 individuals with MDD (53% female) were included into the study, comprising 51 from Austria and 50 from Japan. As shown in Table 1, the two groups differed significantly in terms of most sociodemographic characteristics. In addition, Austrian MDD patients had a significantly longer duration of illness, and presented with significantly higher symptom severity.

**Table 1.** Sociodemographic and Clinical Variables.

Variable	Austria (*N* = 51) Mean ± SD (min–max)/ *N*^a^ (%) or Mean rank (rank sum)^b^	Japan (*N* = 50) Mean ± *SD* (min–max)/ *N*^a^ (%) or Mean rank (rank sum)^b^	Diffs. *t*(*df*)/χ^2a^/U(*z*)^b^	*p*
Age, years	36.37 ± 12.27 (18–66) Median = 34	47.20 ± 16.87 (21–83) Median = 50	−3.69 (99)	**<.001**
Education, years	13.9 ± 2.79 (8–22)	15.38 ± 2.27 (12–22)	−2.91 (98)	.**004**
Gender^a^				
Female	26 (51%)	28 (56%)	2.09	.352
Male	23 (34%)	22 (44%)		
Diverse	2 (4%)	–		
Marital Status^a^				
Single	38 (75%)	16 (32%)	18.39	**<.001**
Married/stable partnership	10 (20%)	27 (54%)		
Widowed	1 (2%)	2 (4%)		
Divorced	2 (4%)	5 (10%)		
Occupation^a^				
Employed, full-time	11 (22%)	11 (22%)	33.43	**<.001**
Employed, part-time	6 (11%)	5 (10%)		
Protected employment, rehab	7 (14%)	3 (6%)		
Unemployed	–	7 (14%)		
Retired	1 (2%)	2 (4%)		
Housewife/-husband	–	9 (18%)		
In training	7 (14%)			
Sick-leave	18 (35%)	7 (14%)		
Other	1 (2%)	6 (12%)		
Living Condition^a^				
Alone	24 (47%)	12 (25%)	21.86	.**001**
Parents, relatives	3 (6%)	10 (20%)		
Partner, own family	14 (27%)	27 (55%)		
Flat-Sharing	8 (16%)			
Assisted living	1 (2%)			
Homeless	1 (2%)			
Number of hospitalizations^a^				
None	16 (31%)	26 (52%)	13.04	.**005**
One time	14 (28%)	19 (38%)		
2–5 times	18 (35%)	4 (8%)		
>5 times	3 (6%)	1 (2%)		
Symptom severity (MADRS)	25.27 ± 10.09 (2–43)	14.94 ± 11.62 (0–40)	4.78(99)	**<.001**
Social functioning (PSP)	66.76 ± 16.59 (25–95)	64.18 ± 14.35 (30–95)	0.837(99)	.405
Duration of illness (years) Mean ranks Mann-Whitney-*U*-Test^b^	8.3 ± 9.1 (0.06–43.5) 57.71 (2,885.5)	5.2 ± 5.99 (0.33–29) 43.29 (2,164.5)	−2.485	.**013**

*Note*. MADRS = Montgomery Asberg Depression Rating Scale; PSP = Personal and Social Performance Scale; ^a^ *N and χ*^2^ *=* chi-squared test; ^b^ Mean rank (rank sum) and *U*(*z*) = Mann-Whitney-*U*-Test; Diffs = differences; Bold values indicate statistically significant results (*p* < .05, 2-sided).

A comparison of Austrian and Japanese MDD patients regarding perceived stigma, spiritual well-being, resilience, and QoL is given in Table 2. Austrian study participants scored significantly higher on social exclusion and the total SIS score when compared to Japanese participants. The country difference in perceived social isolation (higher in Austria) lost its significance after Benjamini-Hochberg correction, while the perceptions of financial insecurity and internalized shame were comparable between groups.

**Table 2.** Perceived Stigma, Spiritual Well-Being, Resilience, and Quality of Life in Austrian and Japanese MDD Patients.

Scale (Total/ Subscale)	Austria (*N* = 51) Mean ± SD (min–max)	Japan (*N* = 50) Mean ± SD (min–max)	*t*(*df*)	*p*-Value/*p*_BH_-Value
Perceived stigma (SIS) Total score	56.39 ± 13.870	49.92 ± 12.237	2.459(97)	.**016**
Social Exclusion	16.88 ± 5.435	14.22 ± 4.857	2.562(97)	.**012/.048**
Financial Insecurity	6.58 ± 2.886	5.71 ± 2.424	1.615(97)	.110/.147
Internalized Shame	12.86 ± 4.286	12.02 ± 3.467	1.070(97)	.287/.287
Social Isolation	20.07 ± 5.302	17.96 ± 4.822	2.068(97)	.**041**/.082
Spiritual Well-Being (FACIT-Sp NI) Total score	17.42 ± 11.55 (1–44)	22.12 ± 10.53 (2–42)	−2.12(97)	.**037**
Meaning	8.84 ± 4.83 (1–16)	7.9 ± 4.03 (0–15)	1.05(97)	.295/.295
Peace	4.4 ± 3.83 (0–12)	7.9 ± 4.08 (0–16)	−4.4(99)	**<.001/<.001**
Faith	4.18 ± 4.64 (0–16)	6.33 ± 3.67 (0–13)	−2.55(97)	.**012/.018**
Resilience (RS)	105.5 ± 30.74 (43–175)	106.12 ± 29.93 (42–164)	−.107(97)	.915
Quality of life (WHOQOL-BREF) Overall quality of life	20.56 ± 7.88 (8–40)	22.45 ± 7.3 (8–40)	−1.24(97)	.219
Physical health	82.64 ± 20.6 (40–128)	81.88 ± 14.45 (44–112)	0.21(94)	.834/.834
Psychological health	59.65 ± 20.84 (24–100)	68.73 ± 14.69 (32–100)	−2.47(93)	.**015/.06**
Social Relationships	37.11 ± 12.02 (12–60)	39.35 ± 10.5 (12–56)	−.974(94)	.333/.444
Environment	119.83 ± 21.92 (68–160)	112.41 ± 21.92 (60–160)	1.66(94)	.099/.198

*Note*. SIS = Social Impact Scale; FACIT-Sp NI = Functional Assessment of Chronic Illness Therapy – Spiritual Well-Being Non-Illness Version; RS = Resilience Scale; WHOQOL-BREF = World Health Organization (WHO) Quality of Life Questionnaire – short version; Bold values indicate statistically significant results (p < .05, 2-sided); pBH-Value = Benjamini & Hochberg Correction (False Discovery Rate).

Significant group differences were found in terms of spiritual well-being, with Japanese study participants scoring significantly higher on the total score as well as on the peace and faith subscales of the FACIT-Sp NI. The two groups were comparable in terms of resilience and most QoL domains, except for the psychological health domain, in which Austrian study participants scored significantly lower than Japanese participants.

### Correlates of Perceived Stigma

Bivariate correlations between perceived stigma and key protective factors, that is, spiritual well-being and resilience, were significantly negative in both samples (Table 3). In the Austrian sample, the strongest associations with large effect sizes were observed between perceived stigma and peace (*r* = −.581, *p* < .001) and the FACIT-Sp NI total score (*r* = −.558, *p* < .001). Social exclusion, internalized shame, and social isolation correlated significantly with almost all spiritual well-being domains and with resilience, showing medium to large effect sizes. In the Japanese sample, in turn, perceived stigma was most strongly correlated with meaning (*r* = −.683, *p* < .001) and the FACIT-Sp total score (*r* = −.664, *p* < .001). In addition, there was a significant correlation between social exclusion, financial insecurity, and social isolation and almost all domains of spiritual well-being as well as resilience (medium to large effect sizes). Internalized shame, however, was negatively correlated with resilience.

**Table 3.** Correlations of Perceived Stigma With Spiritual Well-Being and Resilience in Austrian and Japanese MDD Patients.

Pearson-correlations and *r*-to-*z* transformation		FACIT-Sp NI meaning	FACIT-Sp NI peace	FACIT-Sp NI faith	FACIT-Sp NI total score	RS total score
SIS Social Exclusion	*r* (AT)	−.328*	−.373**	−.224	−.351*	−.374**
	*r* (JP)	−.551**	−.572**	−.251	−.520**	−.425**
	*z*	1.350	1.250	.130	1.010	.29
SIS Financial Insecurity	*r* (AT)	−.091	−.243	−.173	−.188	−.166
	*r* (JP)	−.559**	−.462**	−.355*	−.517**	−.474**
	*z*	2.600	1.210	.950	1.840	1.680***
SIS Internalized Shame	*r* (AT)	−.477**	−.477**	−.328*	−.490**	−.466**
	*r* (JP)	−.273	−.199	−.192	−.248	−.384**
	*z*	−1.150	−1.53	−.7	−1.360	−.48
SIS Social Isolation	*r* (AT)	−.532**	−.621**	−.434**	−.603**	−.470**
	*r* (JP)	−.702**	−.669**	−.557**	−.722**	−.681**
	*z*	1.340	.400	.790	1.030	1.550
SIS Total score	*r* (AT)	−.498**	−.581**	−.391**	−.558**	−.505**
	*r* (JP)	−.683**	−.638**	−.444**	−.664**	−.640**
	*z*	1.390	.44	.31	.82	.97

*Note*. SIS = Social Impact Scale; FACIT-Sp NI = Functional Assessment of Chronic Illness Therapy – Spiritual Well-Being Non-Illness Version; RS = Resilience Scale; AT = Austria; JP = Japan; Bold values indicate statistically significant results (p < .05, 2-sided).

* The correlation is significant at the level of .05 (2-sided). **The correlation is significant at the level of .01 (2-sided). ***The correlation is significant at the level of .05 (1-sided).

Fisher’s *r*-to-*z* transformations were applied to examine cultural differences in correlations. A significant difference was observed in the correlation between financial insecurity and resilience: this was stronger in Japan than in Austria (*z* = 1.68, *p* = .047). However, other comparisons showed no statistically significant cross-country differences.

Table 4 summarizes the correlations between perceived stigma, QoL, and social functioning. In both countries, higher levels of perceived stigma were significantly correlated with lower physical, psychological, and social QoL. Reduced social functioning related to financial insecurity in Austria (*r* = −.291, *p* = .04) and to social isolation in Japan (*r* = −.369, *p* = .009).

**Table 4.** Correlations of Perceived Stigma With Quality of Life and Social Functioning in Austrian and Japanese MDD Patients.

Pearson-correlations and *r*-to-*z* transformation		WHOQOL-BREF Physical	WHOQOL-BREF Psychological	WHOQOL-BREF Social Relationships	WHOQOL-BREF Environment	WHOQOL-BREF Overall satisfaction	PSP
SIS Social Exclusion	*r* (AT)	−.256	−.449**	−.529**	−.316*	−.191	−.194
	*r* (JP)	−.402**	−.443**	−.466**	−.562**	−.437**	−.239
	*z*	.79	−.04	−.4	1.490	1.330	.23
SIS Financial Insecurity	*r* (AT)	−.280	−.067	−.140	−.391**	−.094	−.291*
	*r* (JP)	−.308*	−.413**	−.443**	−.617**	−.257	−.102
	*z*	.15	1.790	1.620	1.480	.81	−.95
SIS Internalized Shame	*r* (AT)	−.442**	−.454**	−.391**	−.410**	−.435**	−.204
	*r* (JP)	−.304*	−.211	−.288*	−.216	−.117	.111
	*z*	−.78	−1.33	−.56	−1.04	−1.68***	−.46
SIS Social Isolation	*r* (AT)	−.485**	−.588**	−.481**	−.449**	−.452**	−.027
	*r* (JP)	−.467**	−.600**	−.498**	−.521**	−.634**	−.369**
	*z*	−.110	.09	.11	.45	1.260	1.74***
SIS Total score	*r* (AT)	−.475**	−.565**	−.543**	−.503**	−.402**	−.210
	*r* (JP)	−.490**	−.554**	−.550**	−.612**	−.507**	−.229
	*z*	.08	−.08	.05	.77	.64	.1

*Note*. SIS = Social Impact Scale; WHOQOL-BREF = World Health Organization (WHO) Quality of Life Questionnaire – short version; PSP = Personal and Social Performance Scale; AT = Austria; JP = Japan; Bold values indicate statistically significant results (p < .05, 2-sided).

* The correlation is significant at the level of .05 (2-sided). **The correlation is significant at the level of .01 (2-sided). ***The correlation is significant at the level of .05 (1-sided).

In the Austrian sample, the strongest negative correlations with large effect sizes were observed between stigma and psychological QoL (*r* = −.565, *p* < .001), followed by social relationships (*r* = −.466, *p* < .001), and environment (*r* = −.503, *p* < .001). In addition, internalized shame and social isolation were strongly correlated with all QoL domains, with medium to large effect sizes observed. In the Japanese sample, in turn, perceived stigma was strongly associated with environment (*r* = −.612, *p* < .001), followed by psychological QoL (*r* = −.554, *p* < .001) and social relationships (*r* = −.550, *p* < .001). Furthermore, medium to large effect sizes were observed in the correlations between social exclusion, financial insecurity, as well as social isolation and all aspects of QoL. The correlation between social isolation and social performance was significantly stronger in Japan (*z* = 1.74, *p* = .041). No other comparisons between countries reached significance, indicating broadly similar patterns of association.

### Predictors of Perceived Stigma

The regression model for the entire sample was statistically significant (*F* (4, 98) = 16.97, *p* < .001) and explained 40% of the variance in perceived stigma (*R*^2^ = .419, adjusted *R*^2^ = .395), indicating a high degree of fit according to Cohen (2013). Among the predictors, only spiritual well-being was a statistically significant individual predictor: *β* = −.417, *t* = −3.18, *p* = .002. Other predictors (resilience, quality of life, and social functioning) did not reach significance in this model, although their inclusion contributed modestly to the explained variance. Detailed models of the implemented multiple regression models (for the full sample and for each country) are presented in Tables 5 and 6.

**Table 5.** Multiple Regression Model (1) Predicting Perceived Stigma From Spiritual Well-Being, Resilience, Quality of Life, and Social Functioning.

Model (1)^b^	*R* ^a^	*R*-squared	Corrected *R*-squared	Standard error of the estimator	Durbin-Watson statistics
Whole sample	.647	.419	.395	10.442	1.688
AT	.598	.357	.300	11.602	1.608
JP	.698	.487	.441	9.152	1.944

*Note*. AT = Austria; JP = Japan.

a Influence variables: (constant). Personal and Social Performance Scale (1–100). Resilience Scale total score. WHOQOL BREF overall satisfaction. FACIT-Sp NI total score.

b Dependent variable: SIS total score.

**Table 6.** Regression Coefficients for Perceived Stigma Predictors (Spiritual Well-Being, Resilience, Quality of Life, and Social Functioning) in Model (1).

Model (1)		Non-std coef.		Std. coef.				95.0% CI	Collinearity statistics	
		Regr. coeff	Std.-error	Beta	*t*	Sig.	Lower limit	Upper limit	Tolerance	VIF
Whole sample	1	79.881	6.238		12.805	<.001	67.494	92.267		
	2	−0.498	0.156	−.417	−3.181	**0.002**	−0.808	−0.187	0.359	2.786
	3	−0.08	0.056	−.171	−1.415	0.16	−0.192	0.032	0.423	2.367
	4	−0.191	0.177	−.108	−1.077	0.284	−0.542	0.161	0.612	1.634
	5	−0.066	0.07	−.077	−0.943	0.348	−0.205	0.073	0.931	1.075
AT	1	83.212	9.236		9.01	<.001	64.609	101.814		
	2	−0.446	0.232	−.371	−1.92	0.061	−0.913	0.022	0.382	2.616
	3	−0.076	0.085	−.169	−0.9	0.373	−0.247	0.094	0.405	2.466
	4	−0.154	0.259	−.088	−0.594	0.555	−0.677	0.368	0.658	1.52
	5	−0.118	0.104	−.142	−1.138	0.261	−0.327	0.091	0.918	1.089
JP	1	80.516	8.788		9.162	<.001	62.805	98.228		
	2	−0.372	0.24	−.32	−1.551	0.128	−0.854	0.111	0.274	3.648
	3	−0.147	0.08	−.324	−1.845	0.072	−0.308	0.014	0.378	2.642
	4	−0.19	0.244	−.113	−0.778	0.441	−0.683	0.303	0.549	1.822
	5	−0.039	0.098	−.046	−0.398	0.692	−0.236	0.158	0.87	1.149

*Note*. Dependent variable: SIS_total score. VIF = variance influence factor. 1 = (Constant); 2 = FACIT_Sp NI_total score; 3 = Resilience Scale total score; 4 = WHOQOL BREF overall satisfaction; 5 = Personal and Social Performance Scale; AT = Austria; JP = Japan; Bold values indicate statistically significant results (*p* < .05, 2-sided).

The separate regression models for both countries were significant (Austria: *F* (4, 49) = 6.26, *p* < .001, adjusted *R*^2^ = .300; Japan: *F* (4, 48) = 10.46, *p* < .001, adjusted *R*^2^ = .441). None of the predictors reached statistical significance, though spiritual well-being in both countries and resilience in Japan showed moderate effect sizes.

After adding age and depression severity in hierarchical multiple regression analyses (Tables 7 and 8), the model significantly improved (*F* (6, 92) = 13.23, *p* < .001) with a total explained variance of 44% (Δ*R*^2^ = .056, *p* = .009). Age became a significant predictor (*β* = −.202, *p* = .015), suggesting that older participants reported lower stigma. Depression severity did not significantly contribute to the model.

**Table 7.** Hierarchical Multiple Regression Model (2): Controlling Predictors of Stigma (Spiritual Well-Being, Resilience, Quality of Life, and Social Functioning) for Age and Depression Severity.

Model (2)^b^	*R* ^a^	*R*-squared	Corrected *R*-squared	Standard error of the estimator	Change statistical values					Durbin-Watson statistic
					Change in *R*-squared	Change in *F*	*df*1	*df*2	∆ or Sig. Change to *F*	
Whole sample	.690	.475	.441	10.031	.056	4.927	2	92	**0.009**	1.810
AT	.647	.419	.338	11.288	.061	2.268	2	43	0.116	1.774
JP	.767	.589	.530	8.389	.101	5.180	2	42	**0.010**	1.805

*Note*. AT = Austria; JP = Japan; Bold values indicate statistically significant results (*p* < .05, 2-sided).

a Influence variables: (constant), social functioning (PSP), resilience (RS), overall satisfaction with quality of life (WHOQOL BREF overall satisfaction), spiritual-well-being (FACIT-Sp NI), age, depression severity (MADRS)

b Dependent variable: perceived stigma (SIS total score).

**Table 8.** Regression Coefficients for the Predictors of Perceived Stigma, Controlled for Age and Depression Severity (Spiritual Well-Being, Resilience, Quality of Life, and Social Functioning) in Model (2).

Model (2)		Non-std coef.		Std. coef.	*t*	*p*	95.0% CI		Collinearity statistics	
		Regr. coef.	Std.-error	Beta			Lower limit	Upper limit	Tolerance	VIF
Whole sample	1	77.813	8.600		9.049	.000	60.734	94.893		
	2	−0.294	0.170	−.246	−1.729	.087	−0.632	0.044	0.280	3.565
	3	−0.088	0.055	−.188	−1.583	.117	−0.198	0.022	0.405	2.471
	4	−0.136	0.180	−.077	−0.753	.453	−0.494	0.222	0.546	1.833
	5	−0.050	0.070	−.058	−0.715	.476	−0.190	0.089	0.855	1.169
	6	−0.176	0.071	−.202	−2.481	.**015**	−0.316	−0.035	0.861	1.161
	7	0.199	0.123	.179	1.621	.108	−0.045	0.444	0.468	2.135
AT	1	104.65	15.14		6.911	.000	74.12	135.19		
	2	−0.346	0.264	−.288	−1.309	.198	−0.879	0.187	0.279	3.585
	3	−0.117	0.088	−.259	−1.332	.190	−0.294	0.060	0.357	2.798
	4	−0.251	0.273	−.142	−0.920	.363	−0.800	0.299	0.564	1.772
	5	−0.158	0.109	−.191	−1.458	.152	−0.378	0.061	0.791	1.264
	6	−0.270	0.145	−.236	−1.855	.070	−0.563	0.024	0.838	1.193
	7	−0.171	0.230	−.125	−0.741	.463	−0.635	0.294	0.473	2.114
JP	1	59.16	10.835		5.46	.000	37.294	81.025		
	2	−0.130	0.237	−.112	−0.546	.588	−0.609	0.350	0.234	4.266
	3	−0.106	0.087	−.234	−1.215	.231	−0.283	0.070	0.265	3.779
	4	−0.043	0.234	−.026	−0.183	.856	−0.516	0.430	0.502	1.992
	5	0.046	0.094	.054	0.489	.627	−0.144	0.236	0.790	1.266
	6	−0.092	0.093	−.127	−0.988	.329	−0.280	0.096	0.594	1.684
	7	0.494	0.161	.465	3.077	.**004**	0.170	0.819	0.429	2.330

*Note*. VIF = variance influence factor. 1 = (Constant); 2 = FACIT-Sp NI total score; 3 = RS total score; 4 = WHOQOL BREF overall satisfaction; 5 = Personal and Social Performance Scale; 6 = age; 7 = Depression severity (MADRS); AT = Austria; JP = Japan; Bold values indicate statistically significant results (*p* < .05, 2-sided)

Adding age and depression severity to the model to control for the predictive role of spiritual well-being, resilience, QoL, and social functioning in perceived stigma did not significantly improve the model. In the Japanese sample, including age and depression severity significantly improved the model (*R*^2^ = .589, adj. *R*^2^ = .530, *F* (6, 43) = 10.26, *p* < .001). Depression severity emerged as the only significant predictor (*β*= .465, *p* = .004), suggesting that a higher degree of depressive symptoms was linked to higher perceived stigma in Japan.

Multiple regression with interaction terms revealed no significant differences in the predictive effects of spiritual well-being, resilience, QoL, age, and depression severity on perceived stigma between Austria and Japan, suggesting comparable effects across contexts. Although resilience and quality of life were significantly associated with perceived stigma in the bivariate analyses, these associations were not significant in the multivariable regression models. This likely reflects the fact that the psychosocial predictors share variance, suggesting that their associations with stigma may depend on broader constructs, such as spiritual well-being.

## Discussion

This study is, to our knowledge, the first transcultural comparison of perceived stigma and its psychosocial correlates in MDD, focusing on spiritual well-being, resilience, QoL, and social functioning. Across both samples, higher stigma was consistently associated with poorer QoL, and spiritual well-being emerged as the strongest protective factor. Differences between countries were also observed: Austrian participants reported higher levels of perceived social exclusion and social isolation, while the correlation between resilience and financial insecurity was stronger in Japan. In regression models, spiritual well-being and age remained significant predictors of stigma, even after controlling for depression severity.

Previous studies have highlighted the pervasive role of stigma in impairing life satisfaction, social integration, and recovery across various mental health conditions (Corrigan & Watson, 2002; Livingston & Boyd, 2010). Research has also demonstrated the protective role of personal resources, such as social support, coping strategies, and spirituality in mitigating stigma-related distress (Koenig, 2015; Livingston & Boyd, 2010). Our findings extend this evidence by showing that spiritual well-being is a particularly robust predictor of lower stigma in people with MDD, across two distinct cultural contexts. Furthermore, the finding that age predicts lower stigma could provide a new perspective on contradictory evidence regarding life-course attitudes (Conner et al., 2010; Mackenzie et al., 2006; Schomerus et al., 2015). Such discrepancies highlight the need for further investigation of generational influences on stigma perception.

Consistent with H1, both spiritual well-being and resilience were negatively associated with perceived stigma, while higher stigma was linked to poorer QoL in both countries. These results are consistent with previous studies demonstrating that stigma undermines well-being and social integration (Corrigan & Watson, 2002; Livingston & Boyd, 2010) and that personal resources such as spirituality and resilience can buffer its impact (Chen et al., 2023; Hofer et al., 2019). Contrary to expectations and previous research, however, no overall correlation between stigma and social functioning was observed (Goldberg, 2011; Livingston & Boyd, 2010). This may indicate that stigma affects functioning in domain- and country-specific ways, as seen in the pooled sample. Alternatively, it may suggest that functional impairment in MDD is more strongly driven by symptom severity, with stigma exerting only indirect effects.

Turning to H2, our data did not reveal any significant differences in the relationship between stigma and psychosocial factors in Austria and Japan. However, several descriptive differences are important to consider. Higher perceived social exclusion in Austria may reflect the greater depression severity, which is known to intensify stigma experiences. (Gaebel et al., 2006). Cultural influences likely also play a role. In more individualistic contexts such as Austria, where autonomy and self-reliance are strongly valued, mental illness may be internalized as personal weakness, thereby increasing self-blame and marginalization (Corrigan, 2004; Corrigan & Watson, 2002; Markus & Kitayama, 2014). In contrast, in collectivist Japan, social belonging and relational harmony may buffer against social exclusion, even if stigma manifests in other ways (Kim et al., 2008; Ma, 2020). The stronger link between resilience and financial insecurity in Japan may reflect the close ties between employment, family expectations, and social status in this setting, where economic difficulties are often associated with shame (Kim et al., 2008; Yang et al., 2007). The urban context may also have influenced the results. The dense urban environment in Tokyo likely provides greater anonymity, higher exposure to mental health information, and broader access to services, all of which can reduce stigma (Pescosolido, 2013). By contrast, Innsbruck, as a smaller regional center, may reflect more traditional and personalized stigma patterns (Henderson et al., 2013; Rüsch et al., 2005). Consequently, stigma perceptions in Japan’s highly urbanized sample may not generalize to rural or conservative regions where help-seeking remains strongly stigmatized (Kurihara et al., 2000). Overall, some aspects of stigma, such as internalized shame, appear to be culturally invariant (Corrigan & Watson, 2002; Yang et al., 2007). However, other related dimensions appear to be more sensitive to sociocultural norms, living conditions, and structural care environments.

With respect to H3, spiritual well-being, resilience, and QoL together explained a substantial proportion of the variance in perceived stigma (40% across the full sample). Although resilience and QoL were significantly associated with perceived stigma at the bivariate level, neither remained a significant independent predictor in the regression models. This finding suggests that these variables share variance with other psychosocial factors, particularly spiritual well-being, which emerged as the strongest significant predictor, underscoring its central role in coping with stigma among people with MDD. This aligns with evidence that spiritual well-being enhances resilience, fosters meaning making, and buffers the psychological impact of stigmatization (Chen et al., 2023; Hofer et al., 2019; Sawab et al., 2024). Consequently, resilience and QoL may indirectly contribute to experiences of stigma through broader mechanisms of meaning-making, coping, and psychological adaptation, rather than exerting unique effects.

The model remained robust after adjusting for age and depression severity, increasing explained variance to 44%, with age also predicting lower stigma. This finding contributes to the ongoing debate about whether stigma increases or decreases with age. Over time, individuals may develop stronger coping strategies and greater self-acceptance, becoming less sensitive to social evaluation (Conner et al., 2010; Mackenzie et al., 2006). However, cohort effects may also play a role, as different generations may have different attitudes toward mental illness and may be more or less willing to internalize stigma (Pescosolido et al., 2010; Schomerus et al., 2015). Thus, lower stigma among older participants may reflect individual adaptation and broader generational differences in how mental illness is perceived and experienced. This finding underscores the importance of longitudinal research in distinguishing between developmental and cohort influences.

Although model fit was somewhat stronger in Japan than in Austria, interaction analyses indicated no significant moderation by country. This suggests that the protective role of psychosocial factors such as spirituality may be broadly consistent across cultures. Still, the relatively higher explanatory power in Japan could point to the greater cultural salience of spirituality and communal values in collectivistic contexts, where stronger public stigma and social expectations may make such resources particularly relevant (Ando et al., 2013; Kim et al., 2008; Koenig, 2015; Ma, 2020; Uchino et al., 2023).

The concept of spiritual well-being may carry different meanings in different cultural contexts. This should be considered when interpreting our findings. In more secular Western societies, such as Austria, spirituality is often understood as an individual resource closely associated with creating of personal meaning, existential reflection, and internal coping mechanisms (Koenig, 2015; Pargament et al., 2013). In this context, spiritual well-being primarily functions as an intrapersonal strategy that helps individuals reinterpret illness and reduce self-stigma. In contrast, Japan’s spirituality is embedded in relational and cultural practices, influenced by Buddhist and Shinto traditions that emphasize interconnectedness, acceptance, and harmony (Kim et al., 2008; Ma, 2020; Markus & Kitayama, 2014). Rather than being purely individual, spiritual well-being may be expressed through social roles, implicit beliefs, and practices that support maintaining balance within the group. For individuals living with a diagnosis of MDD, this may facilitate coping by framing suffering as a shared and transient human experience, potentially reducing self-blame while preserving social belonging (Koenig, 2009; Ma, 2020). These differences suggest that, although spiritual well-being appears to be protective in both contexts, the mechanisms through which it operates may vary, reflecting broader sociocultural frameworks rather than a uniform construct.

Our findings have several clinical implications. First, interventions to reduce stigma in MDD should consider strengthening spiritual well-being, which appears to offer protective value across contexts. Second, the observed associations suggest that stigma not only undermines QoL but may also interact with specific psychosocial domains, such as financial insecurity or social exclusion, in ways that differ between cultural settings. This points to the need for tailored psychosocial interventions – such as meaning-focused therapies, resilience-building programs, and community-based supports – that are sensitive to patients’ sociocultural environments.

The strengths of this study include its focus on MDD, the use of validated instruments in two different countries, and the integration of psychosocial predictors such as spirituality and resilience. However, several limitations must be noted, including the cross-sectional design, the modest sample size, and reliance on self-report measures, which may be influenced by cultural response styles, even when translations are validated. While the sample size was sufficient for detecting moderate associations and allowing exploratory cross-country comparisons, it may have been too small to detect smaller effects or more subtle interactions. Consequently, nonsignificant findings should be interpreted cautiously and warrant replication in larger, more diverse samples. The differences in context between the urban study center in Japan and the rural study center in Austria could further limit the generalizability of our cross-national comparisons. Specifically, the highly urbanized, densely populated, and culturally diverse environment of the Japanese study center may influence experiences of stigmatization and spiritual well-being differently than the smaller, more regionally rooted Austrian study center. Additionally, cultural constructs such as spirituality may not be directly comparable in these contexts because they are rooted in different sociocultural frameworks and life experiences. Finally, the Austrian cohort that participated in this study was younger and exhibited significantly higher depression severity than the Japanese group. Since stigma is closely linked to symptom burden and illness severity, the higher levels of perceived social exclusion observed in Austria may reflect greater clinical severity rather than purely cultural influences. Although our regression models accounted for depression severity, statistical adjustment cannot fully distinguish clinical effects from contextual ones. Therefore, the cross-cultural differences identified in this study should be interpreted with caution, as they may be influenced by cultural context as well as underlying differences in illness severity.

## Conclusions

Culturally sensitive approaches that address both individual and communal coping resources are needed to mitigate the burden of stigma in MDD. This study demonstrates that stigma is closely tied to QoL in MDD and highlights spiritual well-being as a central protective factor across countries. Although differences in stigma domains between Austria and Japan likely reflect both clinical severity and sociocultural contexts, the overall findings suggest that enhancing meaning, connectedness, and resilience may be universally beneficial. The stronger association between resilience and financial insecurity in Japan underscores the substantial influence of socioeconomic factors on stigma-related experiences. These results imply that incorporating financial and social support interventions into stigma reduction strategies could be beneficial.
